# Assessment of Safety and Functional Efficacy of Stem Cell-Based Therapeutic Approaches Using Retinal Degenerative Animal Models

**DOI:** 10.1155/2017/9428176

**Published:** 2017-08-27

**Authors:** Tai-Chi Lin, Magdalene J. Seiler, Danhong Zhu, Paulo Falabella, David R. Hinton, Dennis O. Clegg, Mark S. Humayun, Biju B. Thomas

**Affiliations:** ^1^Department of Ophthalmology, USC Roski Eye Institute, University of Southern California, Los Angeles, CA, USA; ^2^USC Institute for Biomedical Therapeutics, University of Southern California, Los Angeles, CA, USA; ^3^Department of Ophthalmology, Taipei Veterans General Hospital, Taipei, Taiwan; ^4^Institute of Clinical Medicine, National Yang-Ming University, Taipei, Taiwan; ^5^Stem Cell Research Center, University of California-Irvine, Irvine, CA, USA; ^6^Department of Physical Medicine & Rehabilitation, University of California-Irvine, Irvine, CA, USA; ^7^Department of Pathology, Keck School of Medicine, University of Southern California, Los Angeles, CA, USA; ^8^Center for Stem Cell Biology and Engineering, University of California, Santa Barbara, CA, USA

## Abstract

Dysfunction and death of retinal pigment epithelium (RPE) and or photoreceptors can lead to irreversible vision loss. The eye represents an ideal microenvironment for stem cell-based therapy. It is considered an “immune privileged” site, and the number of cells needed for therapy is relatively low for the area of focused vision (macula). Further, surgical placement of stem cell-derived grafts (RPE, retinal progenitors, and photoreceptor precursors) into the vitreous cavity or subretinal space has been well established. For preclinical tests, assessments of stem cell-derived graft survival and functionality are conducted in animal models by various noninvasive approaches and imaging modalities. *In vivo* experiments conducted in animal models based on replacing photoreceptors and/or RPE cells have shown survival and functionality of the transplanted cells, rescue of the host retina, and improvement of visual function. Based on the positive results obtained from these animal experiments, human clinical trials are being initiated. Despite such progress in stem cell research, ethical, regulatory, safety, and technical difficulties still remain a challenge for the transformation of this technique into a standard clinical approach. In this review, the current status of preclinical safety and efficacy studies for retinal cell replacement therapies conducted in animal models will be discussed.

## 1. Introduction

Stem cell-based therapies have shown to restore or rescue visual function in preclinical models of retinal degenerative diseases [1–5] which are built on previous data with transplantation of fetal retinal tissue sheets. This has set a standard what these optimal cells can do [6–9]. Although retinal degenerative diseases such as retinitis pigmentosa (RP), age-related macular degeneration (AMD), and Stargardt's disease differ in their causes and demographics, all of them cause RPE and/or photoreceptor destruction which can lead to blindness [1–5]. Currently, there is no clinically accepted cure for irreversible dysfunction or death of photoreceptors and RPE. Since the retina, like other central nervous system tissue, has little regenerative potential [4, 10], stem cell-based therapies that aimed to replace the dysfunctional or dead cells remain a major hope.

In 1959, a rat fetal retina was transplanted into the anterior chamber of a pregnant rat's eye [11]. Several decades later, dissociated retinal cells or cell aggregates were transplanted into the subretinal space of rats [12–17]. In the 80s, Dr. Gouras demonstrated transplantation of cultured human retinal pigment epithelial cells into the monkey retina. The transplanted cells were identified on the Bruch's membrane by autoradiography [18]. Turner and Blair reported high survival (90–100%) and development of lamination for newborn rat retinal aggregates grafted into a lesion site of an adult rat retina [19]. Silverman and Hughes were the first one to isolate stripes of photoreceptor sheets from the postnatal and adult retina [20], and this method was modified later on by other researchers by transplanting photoreceptor sheets [21], full thickness fetal [6, 7, 22–24] or adult retina [25]. These earlier transplantation studies helped to establish “proof of concept” for future cell replacement therapies in the eye. Although the initial transplantation studies did not show any safety issues, ethical restrictions and absence of suitable animal models for preclinical evaluations delayed further progress of this approach [3]. In 2009, human embryonic stem cell- (hESC-) derived RPE cells were transplanted into Royal College of Surgeon (RCS) rats in preclinical studies [26] that eventually lead to clinical trials. Although the long-term outcomes of the preclinical investigations are not yet concluded [27–31], recent advancement in the area of induced pluripotent stem cell- (iPSC-) derived products provided a new source for transplantation. This method uses mature cells that return to a pluripotent state similar to that seen in embryonic stem cells [32–35]. Preclinical testing of iPSC-derived RPE (iPSC-RPE) cells has been established [36, 37], and human clinical trials based on iPSC-RPE have been initiated [38]. These studies indicate survival of the transplanted RPE with signs of visual functional improvement and no signs of adverse events. However, one of the first human clinical trials using autologous iPSC-RPE cells lead by Masayo Takahashi was halted for a period of time after unexpected chromosomal abnormalities were found in the second patient [39, 40]. In a different incident, severe vision loss was observed in three AMD patients after intravitreal injection of autologous adipose tissue-derived “stem cells” (https://blog.cirm.ca.gov/2017/03/15/three-people-left-blind-by-florida-clinics-unproven-stem-cell-therapy/comment-page-1/). The above report raises some concerns regarding the existing safety requirements and regulations of the use of unregulated stem cell trials [41].

In this review, current progress in stem cell-based therapies will be discussed based on safety assessments and functional evaluations conducted in various animal models of human retinal degenerative diseases.

## 2. Stem Cell Sources and Their Applications in the Eye

Stem cell-based therapy for RPE replacement has been initiated at various centers. Since Klimanskaya et al. developed the original protocol for hESC-derived RPE-like cells [42], various groups have used several strategies to derive RPE cells from stem cells. In earlier studies, subretinal transplantation of hESC-derived RPE (hESC-RPE) cells based on cell suspension injection was shown to rescue degenerating photoreceptors and improve vision in immunosuppressed RCS rats [26, 43]. In a more recent technique, a pregenerated RPE monolayer grown on a scaffold and transplanted in immunosuppressed RCS rats showed improved survival of hESC-RPE and better clinical outcomes [44, 45] suggesting that RPE function is dependent on polarization of the transplanted RPE cells and the monolayer morphology [44–46]. iPSCs are considered to have several advantages over hESCs including protection from immune rejection, wide variety of potential sources, and reduced ethical concerns [47]. Transplantation of iPSC-RPE [37, 48] and iPSC-derived photoreceptor precursor cells [49] has demonstrated success in different animal models. The iPSC-RPE cells were shown to have morphological and functional similarities to developing and mature RPE cells *in vitro* and *in vivo* [37, 50–52]. Although it will be advantageous to use patient-derived RPE (autologous transplants), the time requirements and production cost make allograft transplantation a desirable option [47].

Patients need to have a sufficient number of surviving, functional photoreceptor cells; otherwise, replacement of only RPE will not help to rescue vision. Therefore, stem cell-derived photoreceptors [53–56] or retinal progenitor cells (RPCs) have been used with or without RPE for transplantation experiments [3, 57–60]. Previously, several types of scaffolds made of materials having different architectures, biocompatibility, size, and stiffness have been used to enhance cell survival, migration from the scaffold pores, integration into the host retina, and in vivo differentiation [61–63].

Studies have shown that the beneficial effect of RPC transplantation is likely achieved by their differentiation into functional retinal cells and subsequent replacement of lost or dysfunctional elements [64, 65]. Other investigations suggested that success of RPC transplantation is achieved through trophic factor release rather than direct replacement of the lost cells [59, 66–68]. A major challenge in incorporating photoreceptors and other neuronal cell types is the establishment of synaptic connections with the proximal neuronal elements of the recipient retina [2, 69–71]. Using transsynaptic tracing techniques and donor cell label, synaptic connections between fetal retinal sheet transplants and the host retina have been previously reported [72–74]. Replacement therapies involving the whole retina are also in progress using retinal organoids (3D retina) [70, 71, 75, 76]. Recently, hESCs and iPSCs were differentiated into optic cups and storable stratified neural retina [77, 78]. Such 3D retinal tissue derived from iPSCs or hESCs when transplanted in rd1 mice [70, 71] and immunosuppressed retinal degeneration (RD) monkeys [75] developed a structured outer nuclear layer and showed signs of synaptic formations [70, 71, 75]. The above milestone studies highlight the new concepts of regenerative medicine in retinal therapeutics emphasizing the possibility of establishing functional connections between the transplant and the host tissue.

## 3. Animal Models for Stem Cell-Based Therapies

Retinal degenerate rodent models have been extensively used for biomedical research, but because of the key differences between the rodent and human eye, rodent models do not completely replicate the human disease conditions. Most importantly, the rodents do not have a fovea, and in most of the rodents, the photoreceptors are mainly rod cells. Among rodent models, there are both naturally occurring [79–83] and transgenic animal models [84–87]. Light damage [88], laser-induced choroidal neovascularization [89], and retinotoxic agents such as sodium iodoacetate [90] and N-methyl-N-nitrosourea [91, 92] have been also used to induce retinal degenerative conditions. Among this, sodium iodate (SI) has been widely used to induce outer retinal degeneration in otherwise normal animals [93–96].

Rabbits, cats, dogs, pigs, and nonhuman primates have an eye diameter more or less similar to the human eye which allows easy testing of surgical tools and procedures developed for human patients. However, in these large animal models, inducing a disease condition similar to human patients is challenging mostly because the etiology of human diseases is multifactorial, involving both genetic and environmental contributions [1, 2]. The following section summarizes the small and large animal models that are currently used in stem cell-based research.

### 3.1. Mouse Models

The advantage of using mouse models is their ability to express gene mutations mimicking those identified in humans. However, dissimilarities in life span and rate of disease progression between mice and humans limit the interpretation of the disease conditions. A variety of mutations in mice can cause loss of photoreceptors and reduced rod function and hence were used as AMD models [97–103]. In humans, mutations in the Abca4 gene result in Stargardt's disease, RP, cone-rod dystrophy, and the accumulation of lipofuscin granules in RPE, a characteristic of AMD [104, 105]. Therefore, Abca4 knockout mice which also show lipofuscin accumulation in RPE are considered a model for macular dystrophy conditions [106–110]. Mutations of the RPE65 gene in humans cause most frequently Leber congenital amaurosis, with a small percentage of severe early childhood onset retinal dystrophy [111]. Hence, Rpe65 knockout mice are a model for studying RPE65-mediated retinal dystrophy [112, 113]. Transgenic mice with a rhodopsin Pro23His (P23H) mutation that causes photoreceptor degeneration are highly comparable to human RP disease [99, 114, 115]. In humans, a gene responsible for the autosomal dominant form of Stargardt's disease was identified recently [116, 117]. Transgenic mice harboring this defective gene (Elovl4) are considered a good model for macular degeneration diseases because of the accumulation of high levels of lipofuscin in the RPE and subsequent photoreceptor degeneration in the central retina. This disease pattern closely resembles human Stargardt's disease and AMD [118]. Finally, there are naturally occurring mutations in mice that are used as models of RP disease inheritance [79–83]. Many of the mouse models discussed here are tested for stem cell therapies using cell suspension injections [89, 119]. In conclusion, the wide variety of gene manipulated mouse models provides a valuable tool for studies on therapeutic intervention of various forms of human RD. However, because of their small eye size, implantation of laminated sheets is found to be difficult in mice [44, 120].

### 3.2. Rat Models

Rats' eyes are twice the size of mouse eyes [121] which makes it easier to perform surgical procedures [121] and transplant both fetal retinal sheets [3, 9] and RPE cells grown as a monolayer [44, 45]. The RCS rat is an animal model widely used for investigating therapeutic applications in the eye [122, 123]. The dystrophic RCS rats are characterized by RPE dysfunction due to the deletion in the Mer tyrosine kinase (Mertk) receptor that abolishes internalization of shed photoreceptor outer segments by RPE cells [124]. Accumulation of debris in the subretinal space can lead to drastic photoreceptor degeneration and rapid loss of vision. In RCS rats, the degeneration progresses slowly. At one month of age, the retinal thickness remains close to the normal level [125] and near complete photoreceptor layer thickness is present [126]. Subretinal transplantation of RPE cells derived from both iPSC [127] and hESC [44, 128–130] into 21 to 28-day old RCS rats showed photoreceptor preservation and rescue of declining vision. Certain other rat models mimic the pathology and progression of RD such as the OXYS rat which spontaneously develops a phenotype similar to human aging and AMD-like pathology [131, 132]. The transgenic P23H rat (available in 3 lines with different degeneration rates), similar to P23H mouse, is frequently used as a model of studying RP diseases [85, 133]. S334ter rats carry a mutant mouse rhodopsin which leads to photoreceptor degeneration [134–136]. The five lines of this model have different characteristic rates of RD, in which S334ter line-3 and S334ter line-5 represent fast and intermediate slow degenerating models, respectively [87]. Several studies have been performed in the above rat models to assess the feasibility of retinal cell replacement therapies [3, 73, 137–141].

The advantage of using slow degeneration models is that they mimic the generally slow progression of human disease conditions. With the inner retina relatively well preserved, there is better opportunity for rescue or restoration of vision following various treatment strategies [138, 142]. However, challenges like immunological reactions and the presence of residual host photoreceptors can make it difficult to detect the transplant effects. To overcome the immunological issues, recently immunodeficient rat models (more details are provided in Section 4) are developed for testing cell-based therapies [143, 144]. In summary, rodent models are currently the leading *in vivo* tool for testing retinal cell therapies due to their affordable cost and easy availability [145].

### 3.3. Rabbit Models

Rabbits have an eye size comparable to humans and are considered a desirable model to examine therapeutic effects. However, the rabbit retina differs from that of human because it is rod-dominated and contains the visual streak, a horizontal band lying inferior to the optic nerve absent in humans [146, 147]. The densities of rods and cones in the visual streak are higher than elsewhere in the entire retina [146, 147]. Despite this difference, full-field electroretinography (ERG) developed for the human eye can be used in the rabbit with reproducible results [148]. The transgenic TgP347L rabbit closely tracks human cone-sparing RP disease [86, 149]. Histopathological study in TgP347L rabbits reported that the retinal degeneration developed earlier in the visual streak than in other areas [86] along with some ERG abnormalities [150].

Previously, a dose-dependent correlation between the intravitreal injection of sodium iodate (SI) and retinal degeneration (RD) has been reported in rabbits [151]. According to the investigators, since injected SI may not be evenly distributed in the vitreous due to its uneven liquefaction characteristics, uneven retinal degeneration is caused. Another photoreceptor degeneration rabbit model produced by intravitreal injection of N-methyl-N-nitrosourea showed selective but inconsistent photoreceptor degeneration [152]. Subretinally injected hESC-RPE in immunosuppressed SI-induced RD rabbits failed to integrate into the areas that showed geographic atrophy-like symptoms [153]. This shortcoming was probably due to the unique features of the rabbit eye with a higher degree of immune rejection [153]. In summary, rabbits serve as a useful midsized animal model to study human diseases and therapeutics because they have large eyes compared to rodents.

### 3.4. Cat Models

Abyssinian cats with inherited rod-cone degeneration (rdAc model) are used as a model for studying retinal therapeutics [154, 155]. The genetic defect causative of retinal degeneration in Abyssinian cats has been identified as a single base pair change in intron 50 of the centrosomal protein 290 (CEP290) gene (IVS50+ 9T>G). This results in abnormalities in the transport and distribution of phototransduction and/or structural proteins through the connecting cilia resulting in photoreceptor degeneration [156]. A high prevalence of affected and carrier cats (45% and 44%, resp.) in the population was first observed in Sweden in 1983 [155]. The cause is speculated to be inbreeding [157]. In addition to have tapetum lucidum (discussed in the next section) which is different from the human eye, a major shortcoming of this model is that it does not entirely resemble the human RP diseases where the peripheral retina is strongly abnormal compared to the central area that remain relatively less damaged. No such distinction is observed in Abyssinian cats. In this model, the degeneration is evenly distributed during the early stages where normal and diseased photoreceptors are often found side-by-side [158]. In addition, this cat model manifests a very slow progression of degeneration, taking from 12 months up to four years [154, 155, 157]. Cat breeds with faster RD disease conditions are now available [159, 160]. An early onset autosomal recessive RD disease in Persian cats was virtually completed at 16 weeks of age [160]. Another cat model, the CrxRdy cats, develops retinal thinning that initially takes place in the central retina [159]. An acute, reliable, and complete photoreceptor degeneration model in cats can be achieved by ear vein injection of high-dose iodoacetic acid [161]. Retinal sheet transplantation studies conducted in Abyssinian cats showed good signs of transplant integration with the host retina and lamination of transplant photoreceptors. However, no considerable functional improvement was noticed [162].

### 3.5. Dog Models

A major difference in dog eyes from that of humans is the tapetum lucidum, which is a multilayered reflective tissue of the choroid. The tapetum lucidum is interposed between the branching vessels in the choroid and the single layer of the choriocapillaris beneath the retina. The RPE cells over the tapetum lucidum are normally unpigmented. The tapetum lucidum acts to amplify and reflect light back through the photoreceptor layer again in dim light conditions [163]. Tapetum degeneration called toxic tapetopathy has been described in association with the administration of several drugs in beagle dogs [164–168]. Toxic tapetopathy is the characteristic of an altered tapetal color with degeneration or necrosis of the tapetum lucidum [164, 168]. Tapetum degeneration is not observed in the eyes of animals without a tapetum lucidum (rodents, monkeys) [164, 168] and most importantly not in humans [168].

A naturally occurring canine model of autosomal dominant RP caused by a *RHO* mutation was found to strongly resemble the human RP phenotype [169]. High similarity in eye size and preretinal light transmission characteristics between dogs and humans made this model suitable for examining the genetic and environmental causes of RD diseases. Previous studies demonstrated acute retinal injury in *RHO* mutation dogs after exposure to strong light [170, 171]. By varying the dose of light exposure, its long-term consequences including fast or slow disease progression and injury repair have been examined [170]. Although there have been no reports of stem cell-based studies conducted in these animals, this dog model can be considered a suitable candidate for future preclinical studies.

### 3.6. Pig Models

Several transgenic pig models have been developed for RP diseases, including the Pro347Leu transgenic pig with a rhodopsin mutation [84, 172, 173], the P347S transgenic pig [174, 175], and the P23H transgenic pig, which is considered a model for autosomal dominant RP [176]. Disease progression in most of the above models is slow, making it difficult to assess therapeutic benefits.

Several reports of cell transplantation experiments conducted in pigs are available. Rhodopsin transgenic pigs have been used for transplantation of full-thickness retina [177] and retinal progenitor cell (RPC) transplantation effects [178]. A feasibility and safety study of subretinal implantation of an hESC-RPE monolayer has been reported in immunosuppressed Yucatán minipigs [179]. This study demonstrated preservation of the outer nuclear layer and photoreceptor outer segment overlying the implant. In nonimmunosuppressed pigs, adaptive immune responses were activated following allogenic iPSC-RPE transplantation [180]. The above finding suggests that immunologically matched and autologous donor cells should be considered for RPE cell replacement therapies to obviate chronic immune suppression [180].

A major advantage of using pig models is that surgical tools can be developed without much adaptation from the human parameters [1]. Previous RPE cell replacement therapy studies conducted in pigs were focused on testing surgical feasibility of the approach rather than testing functional improvements [1, 181]. This can be also due to the absence of a suitable pig RPE dysfunction model. Although an RPE debridement model can be developed in pigs [182, 183], it is not preferred for testing RPE cell replacement therapies presumably due to both the difficulty in creating a consistent disease pattern and the severity of the trauma that could adversely affect the study outcome.

### 3.7. Nonhuman Primate Models

The macula is a structure of the eye unique to humans, apes, and monkeys that plays a role as the zone of greatest visual acuity. Therefore, nonhuman primates are a potentially valuable animal model for investigating macular diseases of humans [163]. AMD-like appearance could be found in rhesus monkey (*Macaca mulatta*), cynomolgus macaque (*Macaca fascicularis*), and the Japanese macaque (*Macaca fuscata*). This suggests that the pathogenic mechanisms and associated gene variations are common between human and nonhuman primates [184–190]. Induced RD monkey models have been reported based on systemic injection of iodoacetic acid [191] and cobalt chloride [75], fiber optic light-induced retinal damage [192, 193], and focal damage by severe light exposure [193]. However, these models hold one or more adverse features including ethical issues and inability to produce adequately sized lesions [75]. Housing, maintenance, costs, and ethical concerns due to a close evolutionary relationship to humans further make the nonhuman primate models less appealing for stem cell researchers [194, 195].

Immune rejection of allogeneic iPSC-RPE transplants was studied in cynomolgus monkeys (*Macaca fascicularis*) [196]. In a recent investigation, researchers used a cobalt chloride-induced retinal degeneration RP monkey model to demonstrate possible integration of hESC-derived retinal sheets with the host bipolar cells [75]. The above finding demonstrated clinical feasibility of retina sheet transplantation approach and suggests the need for developing new strategies for future clinical applications [75].

## 4. Tools and Approaches for In Vivo Assessment of the Transplants and Their Functionality

The eyes are one of the few paired organs in the body where it is possible that one eye is treated while the contralateral eye will serve as control. The transparent nature of the eye makes the evaluations possible through noninvasive imaging modalities.

### 4.1. *In Vivo* Imaging of Retinal Transplants and Assessment of Disease Status

**Figure 1:**
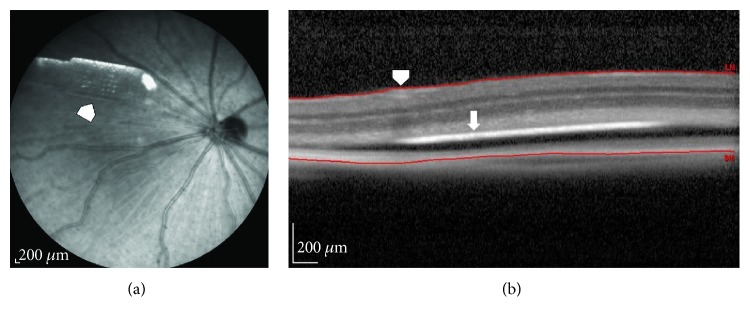
OCT imaging to assess surgical placement of hESC-RPE implantation in rats. (a) Fundus image showing hESC-RPE implants placed inside the rat eye (arrowhead). (b) Optical coherence tomography software was used to measure the distance between the internal limiting membrane (ILM, arrowhead) and the top of the implant (arrow). The maximum and minimum values were recorded. The delta value obtained by subtracting “maximum value − minimum value” can be used to determine if the implant is placed flat or tilted relatively to the retinal surface.

Fundus imaging and fluorescein angiography are used to record baseline and follow-up examinations after stem cell therapies. Optical coherence tomography (OCT) is a noncontact, noninvasive imaging technique widely used in the clinic. The advancement of OCT technology provided rapid assessment of transplant morphology and placement location in the eye [9, 52, 197, 198]. The use of OCT imaging to assess changes in the retinal thickness posttransplantation has been established [9, 197–201]. The above studies conducted in rat models suggested that OCT is a reliable tool for in vivo screening and evaluation of retinal transplants. In our rat experiments, we observed that OCT was helpful using a novel OCT-based screening technique developed by our team. Using OCT software (Heidelberg Spectralis's macular thickness feature), distance between the internal limiting membrane (ILM) and top of the implant was measured (Figure 1). The maximum and minimum values are recorded to determine the delta value. The delta value is obtained by subtracting the “maximum value − minimum value.” Based on the delta value, it is possible to predict whether the implant is placed flat or tilted relative to the retinal surface.

**Figure 2:**
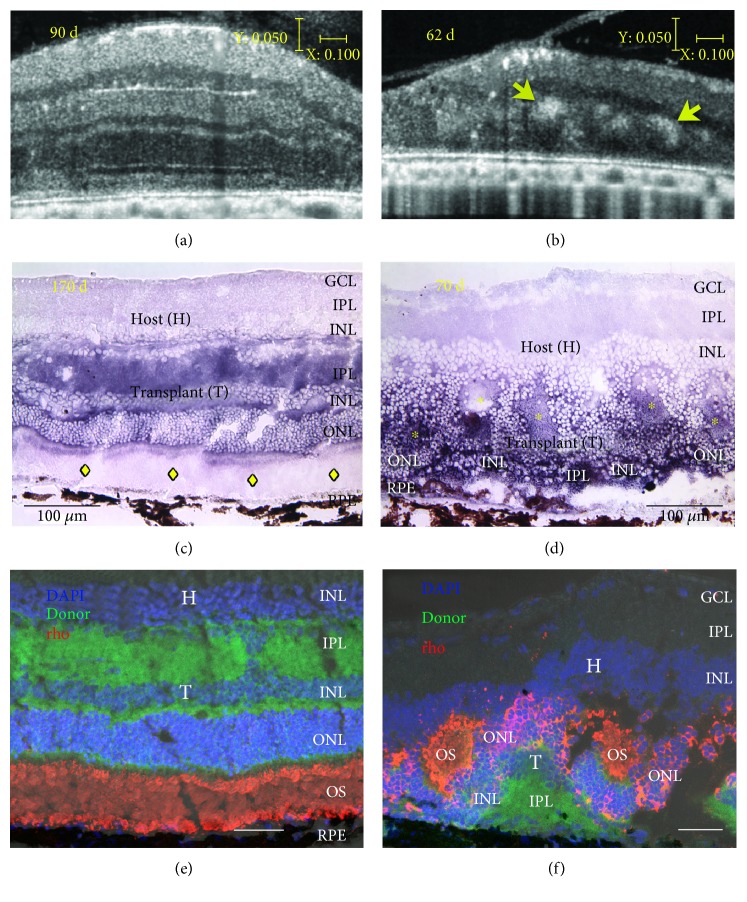
Correlation of live SD-OCT imaging with histology. Fetal retinas (embryonic day 19) derived from rats expressing human placental alkaline phosphatase (hPAP) in the cytoplasm of all cells were transplanted to the subretinal space of immunodeficient retinal degenerate *rho S334ter-3* rats. Transplanted rats' eyes were imaged in vivo by SD-OCT. Two transplant examples are shown. (a, b) Stretched cross-sectional B-scans of laminated (a) and rosetted (b) transplant to better distinguish different retinal layers. Rosettes are indicated by yellow arrows (b) and seen as hyperreflective orbs. (c, d) Transplant-specific histochemistry for hPAP using BCIP (purple). hPAP is expressed in the cytoplasm (not the nuclei) of donor cells. Transplant number 5 (a, c, e) has a large area of lamination parallel retinal layers with photoreceptor outer segments, indicated by yellow diamonds (c) and strong rhodopsin expression (e) in the donor outer retina. Transplant number 1 (b, d, f) is more disorganized with photoreceptors in rosettes [rosette lumens indicated by yellow asterisks in (d)]. The rhodopsin-positive outer segments face inward (f). This transplant (d) was partially placed upside down in the subretinal space. (a, b) Scale bars: vertical bar: 50 *μ*m; horizontal bar: 200 *μ*m; (c, d): 100 *μ*m; (e, f) bars: 20 *μ*m. Modified after Figure 3 of Seiler et al. vision recovery and connectivity by fetal retinal sheet transplantation in an immunodeficient retinal degenerate rat model, IOVS 2017;58:614–630. DOI:10.1167/iovs.15-19028; licensed under the Creative Commons attribution license.

Recently, Seiler et al. [9] used a Bioptigen Envisu R2200 Spectral Domain Ophthalmic Imaging System (Bioptigen, Research Triangle Park, NC, USA) to obtain SD-OCT images of the rat retina that showed similarity between OCT and histology in the lamination pattern and thickness of the transplants (Figure 2). Other techniques like scanning laser ophthalmoscopy (SLO) can generate images from retinal reflectance, autofluorescence, and extrinsic fluorescence. With the confocal arrangement, the SLO is capable of rejecting scattered light, thereby improving image contrast and achieving moderate depth sectioning [202]. Confocal near-infrared SLO imaging was used for *in vivo* detection of subretinally placed hESC-RPE implants in rats [203]. Although the lateral resolution achieved with SLO systems is comparable to that obtained with OCT, the depth resolution was relatively poor. But the advantage of SLO is the ability to detect the presence of pigments on the hESC-RPE *in vivo* [203]. Hence, the survival and potential functionality of an RPE graft can be established. Moreover, SLO is useful when the OCT images are difficult to interpret due to the loss of retinal architecture, as in the case of advanced AMD.

### 4.2. Electrophysiological Assessments

Visually evoked potentials (VEPs) have been used to determine whether the photoreceptor sheet transplants to RD rats can activate the central visual system [204]. VEPs were elicited by using strobe flash stimuli, and responses were recorded contralateral to the stimulated eye. The results showed that the reconstructed retina can produce characteristic light-evoked responses in the visual cortex [204]. Electrophysiological analysis was used to demonstrate that cortical visual function could be preserved by subretinal RPE cell grafting in RCS rats [205]. This was also established using optical imaging techniques [206]. Morphological assessments confirmed good correlation between photoreceptor survival and the extent of cortical functional preservation [206]. However, the degree of visual acuity achieved by transplants cannot be completely addressed using visually evoked cortical responses.

Electroretinography (ERG) is employed to access the diffuse electrical response of the retina. Response to flash ERG has been used to evaluate the visual functional changes in retinal degenerative animal models [43, 207, 208]. ERG assessments have revealed improved photoreceptor function in RCS rats after hESC-RPE injection [43]. A major limitation in using full-field flash ERG is that it may fail to detect signals from the comparatively small transplant area. This is because the ERG response is the cumulative effect of signals from the entire retina, whereas signal output from the transplant area may not be sufficient to generate considerable difference in the ERG wave form [9].

Focal electroretinography (fERG) is used to study a discrete region of the retina and determine if there is significantly more electrical activity in that area compared to the surrounding retina. This technique has been successfully employed in RCS rats to show photoreceptor rescue after iPSC-RPE injection [209]. Although multifocal ERG (mfERG) is also considered an equally efficient tool to analyze focal retinal changes, its application in stem cell research is still not well established. Previously, the technique has been proven to be useful in primate recordings [210] and effective in rats to show focal retinal defects [211]. However, its application in small animal studies is not very popular, presumably due to the inconsistency in the recording pattern which causes difficulties during data interpretation (unpublished observations).

**Figure 3:**
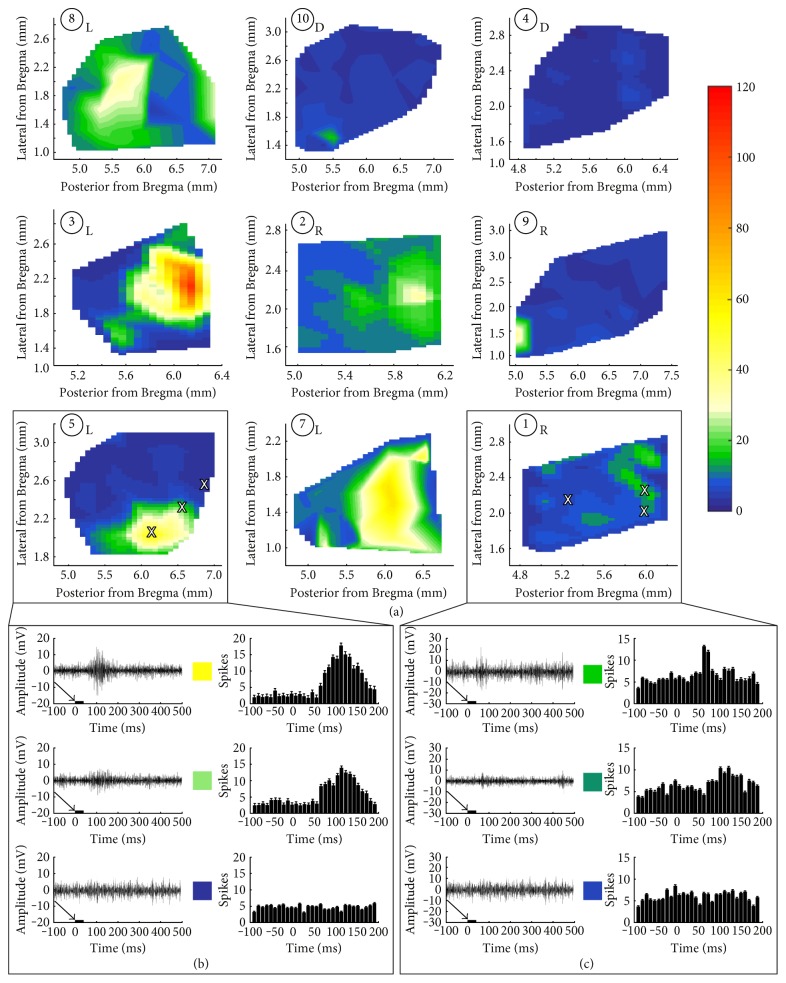
SC recordings from RD nude rats with retinal sheet transplants. (a) Spike count totals (heat maps) over the entirety of the region recorded in SC for all transplanted rats. L: laminated transplant; R: rosetted transplant; D: disorganized transplant. Responses were observed only in certain areas in the SC and were centered on a peak. Sample traces from areas (marked with X) with robust, intermediate, and no response for (b) transplant number 5 with strong responses and (c) transplant number 1 with weak responses. Arrows and black bars indicate the light stimulus. Taken from Figure 7 of Seiler et al., vision recovery and connectivity by fetal retinal sheet transplantation in an immunodeficient retinal degenerate rat model; IOVS 2017;58:614–630. DOI:10.1167/iovs.15-19028; licensed under the Creative Commons attribution license.

Transplant functionality may be reliably assessed by means of electrophysiological mapping of the superior colliculus (SC). The SC receives direct retinal input which corresponds to the areas of the retina that are being stimulated by light [138, 212] and can provide point to point estimates of the retinal function [2]. Our previous studies have demonstrated improved SC responses in rodent models of the RD following cell-based therapies [9, 43, 137, 138, 213]. The SC mapping data can demonstrate that the quality of fetal retinal sheet transplants corresponds to the quality of the SC response [9, 214]. The transplants with more lamination shown in OCT images were later confirmed by SC electrophysiology as having better restoration of visual responses compared to those transplants that were rosetted [9] (Figure 3).

### 4.3. Visual Behavioral Testing

Optokinetic (OKN) testing is a noninvasive visual behavioral testing method widely used for the assessment of spatial visual acuity in rodents [3, 67, 68, 215]. The OKN response is a compensatory eye movement that reduces movement of images across the retina. Factors which affect the OKN responses are the population and distribution of surviving photoreceptors, the inner retina plasticity status, and the morphological status of subcortical visual areas of the brain like the SC [2]. The outcome of stem cell-based therapies can be assessed based on OKN responses by varying the stimulus parameters, such as grating spatial frequency and contrast sensitivity [26, 67, 68, 216]. A major advantage of the OKN testing is the ability to assess visual function without prior training of the animals. It can also enable testing of the left and right eyes independently by using a special apparatus [217] or by changing the direction of the rotation of the stimuli [218]. Previous studies demonstrated that eyes that received stem cell therapies elicit higher levels of optokinetic response compared to the control groups [43, 213, 217, 219, 220]. However, as in the case of full-field ERG, the OKN responses may be inadequate for detecting visual function from a small area showing transplant function [144]. Since the animal could see only a spot-like light from a small area in the visual field, it may fail to evoke head-tracking responses [71]. Another drawback of the OKN testing is its inability to measure higher visual processing since these responses are elicited mostly by subcortical centers. According to McGill et al. [219], since OKN responses in RD animals show conflicting results, it should be used with caution because of the subjective nature of the tests. Other techniques developed for visual behavioral testing include water maze [221, 222] and visual discrimination apparatus [223]. Although the above techniques require extensive training for the animals, they provide the opportunity to test wide variety of visual stimuli that require higher visual processing. However, these tests remain unpopular due to the training requirements, time constraints, and general concerns regarding the accuracy of two-choice tests.

## 5. Safety Studies for Stem Cell Transplantation Approaches

Based on some of the recent reports, the occurrence of adverse events following ocular cell replacement therapies cannot be ruled out. The first study that used autologous iPSC-RPE cells for therapy of AMD in Japan was halted after unexpected mutations were noticed in the iPSCs derived from the second patient. To overcome this issue, cord blood and samples from cord blood banks were targeted as a main source of the cells for reprogramming and a human leukocyte antigen (HLA) homozygous iPSC bank was also established [39]. The major purpose of developing this iPSC bank is to solve the issue of high cost and time consumption in processing autogenic iPSC-RPE cells [224]. There are some reports of human clinical trials based on RPE allografts that failed to survive because of immune rejection [225–227]. The degree of allografts that undergo rejection depends partly on the degree of similarity or histocompatibility between the donor and the recipient. HLA matching has great clinical impact in kidney and bone marrow transplantation but is less of a consideration in heart and lung transplantation [228, 229]. Matching for HLA-B plus HLA-DR resulted in a significant correlation with graft outcome in kidney transplant patients. Grafts with no HLA-B,-DR incompatibilities had approximately 20% higher success rates at one year than grafts with 4 mismatches [230]. Sugita et al. tested all six HLA genotypes (A, B, C, DRB1, DQB1, and DPB1) and reported that the effector T cells can recognize MHC molecules on the allogeneic iPSC-RPE cells, but the immune reaction caused by the T cells can be prevented after HLA blood tests [224]. Therefore, the future clinical trials can make use of allogeneic RPE cells derived from iPSC lines procured from the HLA-homozygous iPSC bank [39, 224]. Nevertheless, further detailed analysis is needed using larger sample size and long-term follow-up [224].

In a recent report, three AMD patients in Florida suffered severe vision loss after receiving injection of autologous adipose tissue-derived stem cells. In this study, adipose-derived stem cells were injected into the eye based on minimal clinical evidence of safety or efficacy. The injection caused ocular hypertension, hemorrhagic retinopathy, vitreous hemorrhage, combined traction and rhegmatogenous retinal detachment, and lens dislocation [41].

**Figure 4:**
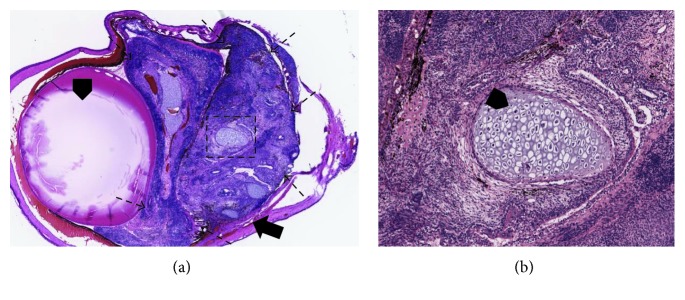
Positive control experiments (injection of undifferentiated cells) conducted in athymic nude rats to show development of tumors in the eye. (a) Teratoma formation at about 6 weeks after injection (2 *μ*l) of undifferentiated hESCE suspension (60,000cells/*μ*l) shown on H&E staining (2x) (arrowhead: lens, arrow: tumor formation originated from the subretinal space, broken arrow: the margin of the tumor). (b) 10x magnification of the black square from (a). The teratoma is composed of various cell types, including cartilage cells (arrowhead).

The major concern of optimum safety and purity of the cells is that the products should be free of undifferentiated cells and should demonstrate the genetic and functional signature of the desired stem cell-derived tissue. Undifferentiated pluripotent stem cells have the capacity to differentiate into all cell types of the three germ layers and may cause tumor formation. Therefore, extensive testing for the absence of tumor formation and cell migration before implantation is crucial [2]. Differentiation into nondesired cell types is a potential threat to the success of stem cell-derived cell therapies. Confirming the purity of stem cell derivations before transplantation is mandatory [231]. In one study, subcutaneous transplantation of iPSCs into immunosuppressed mice resulted in tumor formation, demonstrating the pluripotency of the injected iPSCs and its capability to evade immune detection [232]. The ability of tumor formation is often assessed using tumorigenicity studies in animal models. According to Nazari et al. [2], assessing tumorigenicity potential in immunocompetent animal models can be misleading since the absence of tumor formation might be related to the ability of the host to reject tumorigenic cells before tumors form. However, this can be overcome by using positive controls (injection of undifferentiated cells) that are expected to develop tumors in the target area (Figure 4).

**Figure 5:**
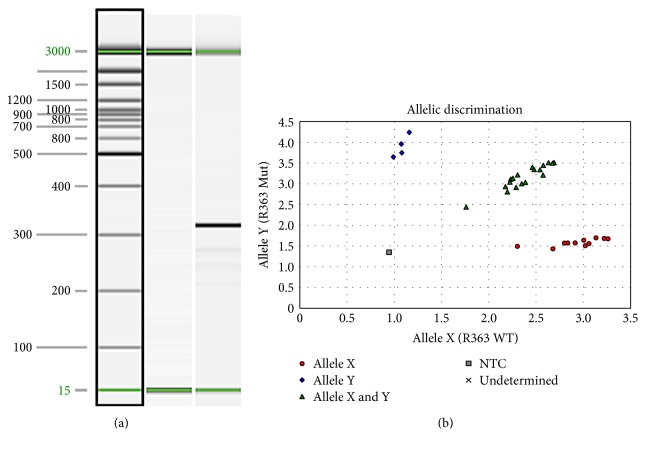
Genotyping assays of immunodeficient S334ter-3 rats. (a) S334ter transgene genotyping assay. Lane 1 15 bp–3 kb size marker. Lane 2 transgene-negative sample. Lane 3 transgene-positive sample. Sizes in base pairs (bp) are indicated to the left of the image. An amplicon of 350 bp indicates the presence of the transgene. The 15- and 3000-bp alignment markers are present in all lanes. (b) Allelic discrimination assay plot for detection of the Foxn1rnu mutation. The fluorescence levels of VIC (wild type, allele X) and FAM (mutant, allele Y) are plotted on the *x*-axis and *y*-axis, respectively. The genotypes of each sample are represented by blue diamonds (homozygous Foxn1rnu), red circles (homozygous for the wild-type Foxn1 allele), or green triangles (heterozygous +/Foxn1rnu). The no template negative control is represented by the gray box. Reprinted from *Graefes Arch Clin Exp Ophthalmol,* vol. 252, Seiler et al., a new immunodeficient pigmented retinal degenerate rat strain to study transplantation of human cells without immunosuppression, pages 1079–1092, copyright (2014), with permission from Elsevier. DOI 10.1007/s00417-014-2638-y.

**Figure 6:**
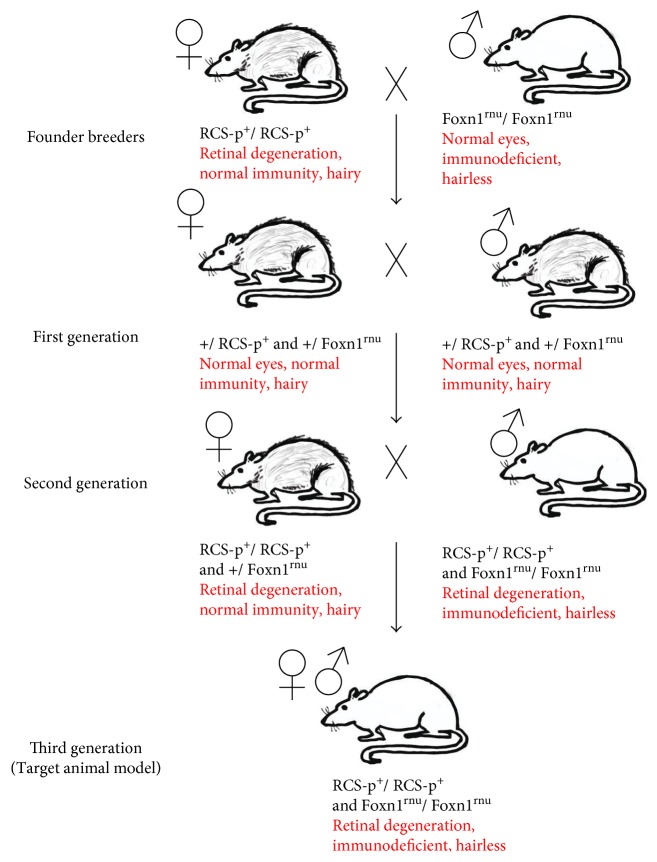
Breeding of immunodeficient RCS rats. Initial mating was performed between male athymic nude rats (Hsd:RH-Foxn1mu) and female dystrophic RCS rats (Mat LaVail, UCSF) to generate F1 pups. The F1 rats were further crossed to generate F2 litters. Pups that are double homozygous (homozygous for RPE dysfunction gene and immunodeficiency gene) were identified based on phenotypic and genotypic expressions.

Although the eye is to a large extent regarded as an immune privileged organ, there is strong evidence for immune response to xenografts [199, 233–235]. When disease models are used for assessing functional efficacy, immunosuppressant drugs are administered to avoid immunological rejection. Most of the preclinical studies involving human-derived cells used animal models that are exposed to severe immunosuppression regimes [26, 196]. Administration of immunosuppressants in rodents is labor intensive and may cause additional pain and discomfort to the animals. A recent study demonstrated more adverse effects of immunosuppression in animal models. Cyclosporine A plus dexamethasone-administered RCS rats showed depressed scores on visual behavioral and electrophysiological testing [236]. To overcome the above issues, we have developed new immunodeficient rat models. This was accomplished by crossing between nondystrophic immunodeficient animals (NIH nude rats) and RD disease models. The double homozygous pups (immunodeficient RD) can be determined by genotyping [143, 144]. Based on this, an immunodeficient S334ter-line-3 rat colony has been established which eliminated the need for immunosuppression when transplanting xenografts [143] (Figure 5). More recently, a new immunodeficient RCS rat model has been also created [144] and is currently being tested for various stem cell-based products (Figure 6). By employing these models, it is possible to justify ethical concerns by reducing animal use and the overall study cost can be considerably lowered.

## 6. Conclusion

Stem cell-based therapies provide a new treatment option for retinal degenerative diseases that were previously considered incurable. Preclinical experiments conducted in animal disease models demonstrated functional efficacy and safety of ocular cell replacement therapies. Studies conducted in large animal models helped to establish the surgical techniques required for clinical trials. The above animal studies have paved the way for several clinical trials based on cell-based therapies currently in progress.
